# Letters to Physicians

**Published:** 1910-12

**Authors:** George M. Gould

**Affiliations:** Ithaca, N. Y.


### Letters to Physicians

By GEORGE M. GOULD, M.D.
Ithaca, N. Y.

### I. Differential Diagnosis by Cycloplegia in Headache, Migraine, Gastric Disease, "Neurasthenia," and so forth.

*My Dear Dr. B.*—You are the best physician in two Americas! Didn't know it? Don't know why? Because you have carried out the method of making an infallible exclusion, or differential, diagnosis in the most frequent, life-wrecking, obscure and disputed diseases. These diseases comprise nearly all the worst maladies which afflict civilised people; they are those of which the origin and nature are so unknown, even so unguessed-at, that the whole profession has about agreed to consider them inexplainable and incurable. Incurable and inexplainable they certainly are, upon any theory yet advanced. They are not infectious, plainly, and they are therefore of no interest to the Lords of Pathology. They are the plague of the practising physician because he cannot cure these tormented life-long suffering patients. They have no interest for the vital statistician and political economist because only death and the lethal diseases arouse the attention of these gentlemen, and these diseases kill only by using their effects (the organic and infectious diseases) as their executioners. They do not concern anybody, apparently, except the millions of agonised patients and their immediate friends. These diseases are headache, and sickheadache ("migraine"), functional gastric and intestinal diseases, insanity, epilepsy, "neurasthenia," vertigo, swooning, and a dozen or two more such conditions named, or misnamed, or unnameable.

There has recently arisen a small class of physicians who aver that these diseases are often due to the eyestrain of ametropia. The great mass of the physicians of the world are either entirely innocent of the knowledge of such a theory, or they disbelieve in it, or they are too scornful of it even to speak of it. The majority have found that their oculists also deny it, and prove the truth of their disbelief by failing to cure such patients with glasses. The situation is thus extremely amusing, or at least it would be if the fact did not prove the utter falsity of the claim of medicine to be "scientific," and if the noncures did not continue the bitterest tragedies of millions of sufferers.

For some twenty years or more I have urged, and reurged, iterated and reiterated, a simple and effective method of settling the controversy. A number of years ago I begged the medical superintendent of a large and exceedingly prosperous, rapidly growing epileptic colony, to allow me to settle, or to aid in settling the vexed question of the cause of epilepsy by the use for a month, in a picked one hundred patients, of cycloplegia. Feeling that the result might throw some light on the nature of this awful disease, or, too "scientific" to care to bother with such tomfoolery, the researcher into pathogenesis roundly refused. During this score of years I have begged my general physician friends to try the plan in cases of migraine, hysteria, neurasthenia, headache, dyspepsia, and the like, and thus if I was correct, be able to renounce the fashionable drugs and other unavailing methods of treatment. I assured them that I had proved it a wholly trustworthy method of differential diagnosis in many thousand cases; that every day for these years I have found cycloplegia stopped these headaches, vomitings, *et id genus omne,* while the eyes were under its influence, the symptoms recurring with greater intensity the hour "the drops" wore off.

The strange, utterly inexplainable thing during all this time, is that not a single physician has tried the easy method of establishing a diagnosis, and of solving the point in dispute, which is the scandal of medicine.

Not one, except you! At last the light of common sense and genuine "scientificalness" has through you begun to spread its light. You had a sound-appearing, sturdy, and even robustly healthy patient, aged thirty-nine, who had sickheadache from early childhood. These headaches had been of late growing more intense and frequent, at last bringing him down twice a week. He had been forced to give up his business two or three days a week for the past summer. An observant physician, such as yourself, could see that this ingravescence was probably due to presbyopia, now coming on and intensifying the previous eyestrain. So little did his former physicians suspect the cause that he was never sent to an oculist until 1898. Headaches, and their associate phenomena kept on. Glasses changed every year since 1904 made no difference. Drugs, and drugs, and more drugs, relieved not the suffering. Prisms were used in his glasses, discontinued, reordered, and so on,—it did not matter. In 1898 the inevitable and customary "nervous breakdown" occurred. After which, consultations, and, of course, a long sea-voyage. Then three months were spent in the woods, where, as he read little or not at all, he felt better. Aspirin sometimes "headed off" his headache when his eyes showed an oncoming attack by "watering" and "sand" in them. Another physician, to be sure, solemnly whispered, "Rheumatism!"

Then you (whom I have never seen, unfortunately for me) wrote me, saying you had heard I had advised the use of atropin-instillations in such cases, as a method of determining whether eyestrain was the cause of the patient's disease. I naturally emphasised my *Yes,* wondering if the crack-o-doom was coming. The drops were used for ten days, but the effects lasted a week or more longer. Not a headache or sign of one took place during these three weeks; the man gained twelve or fifteen pounds during this period, and his "spirits went right up." As the last effects of the cycloplegic were wearing away there came on a violent headache lasting three days.

Could any demonstration be more convincing that the eyes were the source of the man's disease? Either so, or the "brain disease" was reached by the systemic and cerebral effects of the drug, or Fate itself was quelled by the "eye-opener,"—or still more likely, the infinitesimal bit of atropin reached back into the past ages of this patient's ancestors, and extinguished the "iron laws of heredity," to which appeal is made in the pitiful distress of the medical scientists and philosophers.

Well! your patient required the following glasses:

R.+Sph. 1.00+Cyl. 1.12 ax. 90 } Distance
L.+Sph. 0.75+Cyl. 1.12 ax. 90 }

R.+Sph. 1.62 & Cyl. } Near
L.+Sph. 1.37 & Cyl. }

He has no imbalance of the external ocular muscles; he has no disease of the eyes; his visual acuteness is perfect. Of course, correct spectacles must do what the ocular atropinisation did. The trouble seems to be that the exact neutralisation by spectacles of the ametropia is a difficult and rare occurrence. This gentleman has up to now had no recurrence of his former symptoms.

